# Recombinant Antigens Based on Non-Glycosylated Regions from RBD SARS-CoV-2 as Potential Vaccine Candidates against COVID-19

**DOI:** 10.3390/vaccines9080928

**Published:** 2021-08-20

**Authors:** Leandro Núñez-Muñoz, Gabriel Marcelino-Pérez, Berenice Calderón-Pérez, Miriam Pérez-Saldívar, Karla Acosta-Virgen, Hugo González-Conchillos, Brenda Vargas-Hernández, Ana Olivares-Martínez, Roberto Ruiz-Medrano, Daniela Roa-Velázquez, Edgar Morales-Ríos, Jorge Ramos-Flores, Gustavo Torres-Franco, Diana Peláez-González, Jorge Fernández-Hernández, Martha Espinosa-Cantellano, Diana Tapia-Sidas, José Abrahan Ramírez-Pool, América Padilla-Viveros, Beatriz Xoconostle-Cázares

**Affiliations:** 1 Department of Biotechnology and Bioengineering, Centro de Investigación y de Estudios Avanzados (CINVESTAV), Av. Instituto Politécnico Nacional 2508, México City 07360, Mexico; leandro.nunez@cinvestav.mx (L.N.-M.); gabriel.marcelino@cinvestav.mx (G.M.-P.); berecalpe@yahoo.com.mx (B.C.-P.); byvargas@cinvestav.mx (B.V.-H.); olivaresmtz.ana@cinvestav.mx (A.O.-M.); rmedrano@cinvestav.mx (R.R.-M.); dianaa.tapia@cinvestav.mx (D.T.-S.); jramirezp@cinvestav.mx (J.A.R.-P.); 2 Doctoral Program in Nanosciences and Nanotechnology, Centro de Investigación y de Estudios Avanzados (CINVESTAV), Av. Instituto Politécnico Nacional 2508, México City 07360, Mexico; daniela.roa@cinvestav.mx; 3 Department of Infectomics and Molecular Pathogenesis, Centro de Investigación y de Estudios Avanzados (CINVESTAV), Av. Instituto Politécnico Nacional 2508, México City 07360, Mexico; mperezs@cinvestav.mx (M.P.-S.); kbacosta@cinvestav.mx (K.A.-V.); hdgonzalez@cinvestav.mx (H.G.-C.); mespinosac@cinvestav.mx (M.E.-C.); 4 Department of Biochemistry, Centro de Investigación y de Estudios Avanzados (CINVESTAV), Av. Instituto Politécnico Nacional 2508, México City 07360, Mexico; edgar.morales@cinvestav.mx; 5 Laboratory Animal Production and Experimentation Unit, Centro de Investigación y de Estudios Avanzados (CINVESTAV), Av. Instituto Politécnico Nacional 2508, México City 07360, Mexico; battousai_slayer@hotmail.com (J.R.-F.); franco308228727@gmail.com (G.T.-F.); dianapel94@hotmail.com (D.P.-G.); jofernan@cinvestav.mx (J.F.-H.); 6 Transdisciplinary Doctoral Program in Scientific and Technological Development for Society, Centro de Investigación y de Estudios Avanzados (CINVESTAV), Av. Instituto Politécnico Nacional 2508, México City 07360, Mexico; aviveros@cinvestav.mx

**Keywords:** SARS-CoV-2, receptor binding domain, Spike protein, vaccine, viral glycosylation, prokaryotic expression

## Abstract

The Receptor-Binding Domain (RBD) of the Spike (S) protein from Severe Acute Respiratory Syndrome Coronavirus 2 (SARS-CoV-2) has glycosylation sites which can limit the production of reliable antigens expressed in prokaryotic platforms, due to glycan-mediated evasion of the host immune response. However, protein regions without glycosylated residues capable of inducing neutralizing antibodies could be useful for antigen production in systems that do not carry the glycosylation machinery. To test this hypothesis, the potential antigens NG06 and NG19, located within the non-glycosylated S-RBD region, were selected and expressed in *Escherichia coli*, purified by FPLC and employed to determine their immunogenic potential through detection of antibodies in serum from immunized rabbits, mice, and COVID-19 patients. IgG antibodies from sera of COVID-19-recovered patients detected the recombinant antigens NG06 and NG19 (A_450 nm_ = 0.80 ± 0.33; 1.13 ± 0.33; and 0.11 ± 0.08 for and negatives controls, respectively). Also, the purified antigens were able to raise polyclonal antibodies in animal models evoking a strong immune response with neutralizing activity in mice model. This research highlights the usefulness of antigens based on the non-N-glycosylated region of RBD from SARS-CoV-2 for candidate vaccine development.

## 1. Introduction

The Coronaviridae family encompasses several viruses that can cause human diseases ranging from common cold to severe clinical manifestations, such as Middle East Coronavirus Respiratory Syndrome (MERS-CoV), Severe Acute Respiratory Syndrome (SARS-CoV), and COVID-19 (SARS-CoV-2) [1,2]. SARS-CoV and SARS-CoV-2 coronaviruses employ Spike (S) protein to enter the host cells through interaction with the cellular receptor of the angiotensin-converting enzyme (ACE2) [3,4]. S protein is the largest known type-1 viral fusion protein and contains 22 canonical N-glycosylation sites (N-X-S/T, where X ≠ P), as well as 33 O-glycosylation sites [5,6,7,8,9] which could enhance infectivity [10]. This affects the host’s ability to develop an effective adaptive immune response [11], or probably causes epitope masking [12] as has been reported for other viral proteins such as the envelope protein (ENV) of HIV [13], glycoprotein 1 (GP1) of Ebola virus [14], hemagglutinin (HA) of the influenza virus [15], the glycoprotein complex of the Lassa Virus (LASV GPC) [16], and, interestingly, the coronavirus NL63 S protein [17].

SARS-CoV-2 S protein possesses N-glycosylations that can be classified as complex (55%), high mannose content (28%), or hybrid (17%), covering approximately 40% of its surface [18,19]. S protein also has a typical homotrimeric structure of coronavirus, where each monomer consists of a signal peptide (SP), S1 and S2 subunits (the first responsible for receptor binding and the second with an anchoring function to the viral membrane), a transmembrane domain (TM), and an intracellular domain (IC) [20]. The S1 subunit contains the Receptor Binding Domain (RBD) and within it, the receptor-binding motif (RBM). RBD has a total of four disulfide bridges [21] and three N-glycosylation sites: two experimentally verified (N331 and N343), and a third predicted one (N370), according to comparisons with RBD of SARS-CoV. Four O-glycosylation sites (T323, S325, T333, and T345) have also been found experimentally [6,8].

The S protein has been proposed as a key element for the development of antigens with prophylactic potential and for immunodiagnosis, considering the presence of RBD and its role in the entry of the virus into the host cells. If antibodies against RBD could be generated, it would potentially prevent the entry of the virus and allow a humoral and cellular immune response to inactivate the viral particles [22]. Furthermore, the use of full-length SARS-CoV protein S as a protective vaccine, using recombinant modified vaccinia virus Ankara (rMVA), induced a strong inflammatory response in ferret liver tissues [23] and, in macaque models, the production of anti-Spike SARS-CoV IgG in infected lungs is responsible for acute lung injury by skewing the inflammation-resolving response [24]. The use of SARS-CoV RBD as a promising antigen has demonstrated the induction of neutralizing antibodies, as well as recognition by antibodies present in the serum of convalescent patients [3,25]. Currently, pharmaceutical laboratories are focused on the production of different versions of S protein expressed in eukaryotic systems, including RBD-based subunit vaccines against SARS-CoV-2 [26].

Considering that there are no glycosylation sites in the middle and C-terminal regions of RBD and that several neutralizing antibodies have been reported mapping this region, we evaluated the utility of two RBD SARS-CoV-2 non-glycosylated regions expressed in *Escherichia coli*. Using these antigens for rabbit immunization assays, we were able to detect high titers of IgGs, as well as to detect IgGs in sera from COVID-19 patients. These peptides are proposed for the design of vaccines and therapeutic applications.

## 2. Materials and Methods

### 2.1. Bioinformatic Analysis

The structural model of the open state of S protein (PDB: 7JZL), the sequence of the receptor binding domain (RBD) [27] and the experimental glycosylation data [5], were employed for the selection of the non-glycosylated region of the RBD, from S371 to F541 residues. Furthermore, a second peptide was selected within the Receptor Binding Motif (RBM) [21] which comprises S438 to N487. These antigens will be referred as NG06 and NG19, as Non-Glycosylated proteins of 6 and 19 kDa. Furthermore, we assayed the KWCEC adjuvant, translationally fused to the carboxyl-terminal region of the NG19 to generate NG19m [28].

The physicochemical properties were calculated with ProtParam tool from the ExPAsy server (https://web.expasy.org/protparam/) (accessed on 20 April 2020) [29]. The prediction of antigenic sequences was determined using the Predicting Antigenic Peptides Tool (http://imed.med.ucm.es/Tools/antigenic.pl) (accessed on 20 April 2020) [30]. Potential allergenicity was determined using AllerTOP v2.0 (http://www.ddg-pharmfac.net/AllerTOP/) (accessed on 20 April 2020) [24] and the mapping of IgE epitope module from the AlgPred server (https://webs.iiitd.edu.in/raghava/algpred/index.html) (accessed on 20 April 2020) [31]. The secondary structure of the antigens was predicted using PSIPred Protein Sequence Analysis Workbench (http://bioinf.cs.ucl.ac.uk/psipred/) (accessed on 21 April 2020) [32,33], while the prediction of tertiary structure was predicted *de novo* using trRosetta (https://yanglab.nankai.edu.cn/trRosetta/) (accessed on 21 April 2020) [34], refined, if necessary, with Galaxy Refine Server (http://galaxy.seoklab.org/cgi-bin/submit.cgi?type=REFINE) (accessed on 22 April 2020) [35], validated by using PROCHEK v.3.5 (https://servicesn.mbi.ucla.edu/PROCHECK/) (accessed on 23 April 2020) [36] and ProSA (https://prosa.services.came.sbg.ac.at/prosa.php) (accessed on 23 April 2020) [37]. Representative models were visualized using the UCSF Chimera [38]. For the prediction of oligomerization models for NG19m antigen the GalaxyHomomer software was employed (http://galaxy.seoklab.org/cgi-bin/submit.cgi?type=HOMOMER) (accessed on 18 February 2021) [39].

### 2.2. Molecular Cloning

The vector pUC57 eRBD (SARS-CoV-2) RBD plasmid was employed as a template. It encodes the RBD of SARS-CoV-2 with an optimized codon usage for *E. coli* expression. Oligonucleotides were designed including the 5′-NcoI and 3′-HindIII restriction sites (Table S1) for further cloning. The PCR was performed in a final volume of 25 µL with 15.4 µL of sterile H_2_O Milli-Q, 2.5 µL of Buffer Ex Taq 10X, 2 µL of dNTPs (2.5 mM each), 2 µL of each primer (10 µM), 0.1 µL of TaKaRa Ex Taq Polymerase and 1 µL of plasmid DNA (10 ng/µL). The PCR conditions were initial denaturation at 94 °C for 3 min, followed by 30 cycles at 94 °C for 30 s, alignment at 60 °C for 30 s and extension at 72 °C for 30 s, with a final extension at 72 °C for 10 min. The PCR products were ligated into pCR8/GW/TOPO^®^ (Invitrogen, Waltham, MA, USA), transformed into *E. coli* MACH1-T1 cells and subsequently subcloned into the pCri1a (for NG06 and NG19) or pCri8a (only for NG19m) vectors [40] by digesting 3 µg of each plasmid, purifying the fragments of interest with Zymoclean Gel DNA Recovery Kit (Zymo Research, Irvine, CA, USA) and ligated at a 1:10 molar ratio (vector: insert) with the DNA rapid ligation kit (Thermo Fisher Scientific, Waltham, MA, USA) using 100 ng of vector. The ligation mixture was transformed into *E. coli* MACH1-T1 chemically competent cells by heat shock and incubated on Luria-Bertani (LB) plates with kanamycin (50 µg/mL) at 37 °C overnight. The resulting clones were analyzed by endpoint PCR, restriction, and Sanger sequencing (Macrogen Inc., Seoul, Korea).

### 2.3. Protein Expression and Purification

*E. coli* BL21 (DE3) harboring pCri-1a/NG06 or pCri-1a/NG19 were inoculated in TB broth (1.5% inoculation volume *v/v*) supplemented with kanamycin sulfate (50 μg/mL) and glucose (5 g/L). The cultures were incubated to 37 °C, 200 rpm and induced with 1 mM IPTG (Sigma-Aldrich, Saint Louis, MO, USA) at OD_600 nm_ = 0.7–0.8. Induction culture conditions were for MBP-NG06, 37 °C for 5 h and for MBP-NG19, 18 °C for 16 h. Each gram of biomass was resuspended in 10 mL of buffer A (20 mM NaH_2_PO_4_, 0.5 M NaCl, 20 mM Imidazole, 1 mM PMSF, 1 mM benzamidine hydrochloride, pH 7.4) and lysed by ultrasonication in an ice bath using an Ultrasonic Homogenizer 500 at 150 W (4 min, 5 s for ultrasonication and 30 s for rest interval) (Cole-Parmer, Vernon Hills, IL, USA). The lysate was centrifuged at 35,000 rpm for 30 min at 4 °C.

Purifications from soluble fractions of MBP-NG06 and MBP-NG19 were performed using an AKTA pure 25 (GE Healthcare, Chicago, IL, USA) through immobilized metal affinity chromatography (Ni^2+^-IMAC) employing a HisTrap HP 5 mL column (GE Healthcare, Chicago, IL, USA) previously equilibrated with buffer A, at a flow of 2.5 mL/min. The column was washed with three volumes of buffer A and eluted through a linear gradient (0–100%) of buffer B (20 mM NaH_2_PO_4_, 0.5 M NaCl, 500 mM imidazole, 1 mM PMSF, 1 mM benzamidine hydrochloride, pH 7.4) in 20 column volumes. Subsequently, the protein fractions were exchanged into buffer C (1 mM reduced glutathione, 1 mM oxidized glutathione, 25 mM Tris-HCl, 150 mM NaCl, pH 7.4); using a HiPrep 26/10 Desalting column (GE Healthcare, Chicago, IL, USA). The desalted fractions were combined, tobacco etch virus (TEV) protease was added (1:100 ratio), and the reaction mixture was incubated at 4 °C for 16 h. After cleavage, the samples were purified again using the HisTrap HP 5 mL column. The unbound fraction, which corresponds to the recombinant protein without fusion partner, was concentrated to 0.5 mL with an Amicon Ultra-15 Centrifugal Filter Unit (MWCO = 3000 kDa) (Millipore, Burlington, MA, USA) and loaded onto Superdex 75 10/300 GL column (GE Healthcare) in order to perform a size exclusion chromatography (SEC), which was equilibrated with 2 CV of buffer D (1X PBS) at a flow rate of 0.5 mL/min. For the purification of NG19m antigen in the pCri8a/NG19m vector, we carried out the purification and refolding of inclusion bodies according to a previously described workflow [41] with the following modifications: buffer B with 1 M hydrochloride guanidine instead of 2 M, buffer C with 1% Triton X-100 instead of 1% Triton X-114, and solubilization buffer with 1 mM DTT instead of 1 mM β-mercaptoethanol. All purification steps were analyzed by Tricine-SDS-PAGE at 12% and quantified using the Pierce™ BCA™ kit (ThermoFisher, Waltham, MA, USA).

### 2.4. Immunizations and Serum Sample Processing

New Zealand rabbits (*n* = 2, age = 8 weeks, and weight = 1.93 and 1.90 kg), we immunized with 200 μg of purified antigens NG 06 and NG19, combined with TiterMax Gold Adjuvant (Sigma-Aldrich) (1:1). For Balb/c mice immunizations (*n* = 15, age = 6–8 weeks, and average weight 20 g) 20 μg of NG19m were employed in combination with Rehydragel IV (Chemtrade Solutions, Toronto, Canada) (1:10) to achieve a formulation like that would be used in clinical trials. All the experimental animal models were housed at 12 h light/dark cycles at 20 ± 1 °C with food and water *ad libitum*). The immunizations were delivered through subcutaneous and intramuscular route for rabbit and intramuscular for mice. Booster doses (200 ug) were given at 15 dpi.

Peripheral blood samples were obtained 15 and 30 dpi, as well as from recovered COVID-19 patients. We consider as recovered COVID-19 patients those who did not show disease symptoms 2–4 weeks after a RT-qPCR positive result. RT-qPCR was carried out according to the Berlin Protocol for the detection of SARS-CoV-2 [42]. For cloth formation, collected blood samples were incubated at room temperature for 1 h and then centrifuged at 2000 rpm for 5 min. Sera from recovered COVID-19 patients were heat-inactivated at 56 °C for 30 min to minimize exposure to SARS-CoV-2 in further evaluations.

### 2.5. Western Blot Analyses

Recombinant proteins (15 μg/well) were separated in 8% Tricine SDS-PAGE gels and transferred to polyvinylidene difluoride (PVDF) membranes at 30 V overnight. Membranes were blocked with 5% of non-fat dry milk for 2 h and incubated overnight with rabbit hyperimmune sera (1:1000). The next day, membranes were washed with PBST and incubated for 4 h with a goat anti-rabbit IgG peroxidase-coupled secondary antibody (1:40,000) (Sigma). All the membranes were revealed using ECL^TM^ Prime Western Blotting Detection Reagent (GE Healthcare) following the manufacturer’s recommendations.

### 2.6. Antibody Titration by ELISA

Nunc-Immuno MicroWell 96 well solid plates (Sigma-Aldrich) were coated with 100 µL (0.05 µg/well) of native RBD, NG06, or NG19 proteins diluted in 0.05 M carbonate-bicarbonate buffer pH 9.6 (Sigma-Aldrich) and incubated overnight at 4 °C. To assess specificity detection, respiratory, human pathogen extracts were employed using the NATtrol™ Respiratory Panel 2 (RP2) Controls (Zeptometrix Corporation, Buffalo, NY, USA) respiratory pathogens panel. Plates were blocked with 100 µL of 1% Bovine Serum Albumin in PBS for 1 h at 37 °C and then washed three times with 200 µL of PBST (Phosphate Buffer Saline 1X + 0.05% Tween-20). Sera samples were diluted in PBST (1:50–1:10^6^ for animal models samples and 1:5000 for human samples) employing 100 µL/well and incubated for 1 h at 37 °C. Pre-immune or PCR negative sera were used as negative controls. Plates were washed three times with PBST and secondary antibodies goat anti-rabbit IgG-HRP (Sigma-Aldrich), rabbit anti-mouse IgG-HRP (Sigma-Aldrich) or goat anti-human IgG (H + L), HRP (Thermo Fisher Scientific) were employed at 1:40,000, 1:2000, and 1:5000, respectively. The plates were incubated at 37 °C for 1 h. After washing three times with PBST, 100 µL of 3,3′,5,5′-tetramethylbenzidine (TMB) substrate solution (Sigma-Aldrich) was added, incubating 30 min at room temperature. The reaction was stopped with 100 µL of 0.2 M H_2_SO_4_. The optical density was measured at 450 nm using an Epoch Microplate Spectrophotometer (BioTek Instruments, Winooski, VT, USA). For each sample, the absorbance values obtained were subtracted with the values obtained in the negative controls (wells coated without antigen).

### 2.7. SARS-CoV-2 Surrogate Virus Neutralization Test (sVNT) in Sera of Immunized Mice

The SARS-CoV-2 Surrogate Virus Neutralization Test (sVNT) (GenScript, Piscataway, NJ, USA) was assayed with sera of immunized mice, collected at 30 dpi, according to the manufacturer’s recommendations.

### 2.8. Statistical Analysis

Statistical analysis was performed with GraphPad Prism 6.01 software (San Diego, CA, USA). The difference between the two groups was evaluated using the non-parametric Mann–Whitney ranked test and *p*-value < 0.05 was considered significant. Values obtained with dilution buffer were subtracted from those obtained with the sera.

## 3. Results

### 3.1. Antigen Design Based in a Non-Glycosylated Domain within RDB of Spike Protein

**Figure 1:**
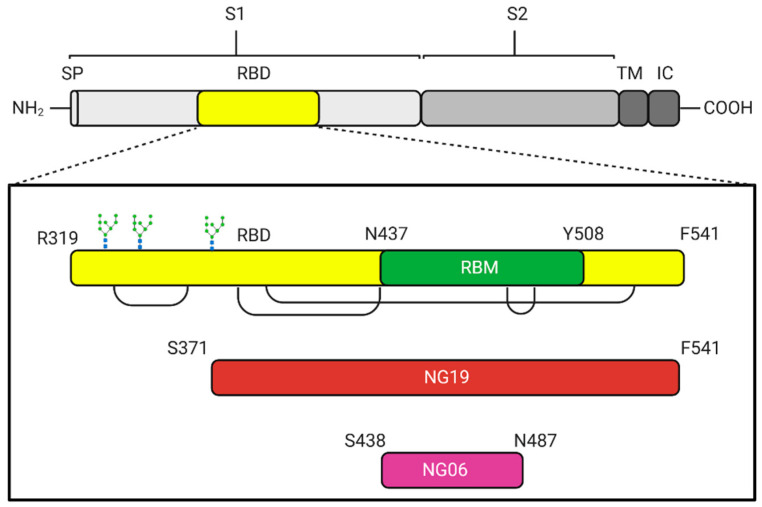
Schematic diagram of the antigens employed as vaccine candidates against SARS-CoV-2, for heterologous expression in the *E. coli*-based platform. SP: signal peptide, S1: Subunit 1, S2: Subunit 2, TM: Transmembrane domain, IC: Intracellular domain, RBD: Receptor binding site, RBM: receptor binding motif, NG19: Non-glycosylated 19 kDa, and NG06: Non-glycosylated 6 kDa. The three glycosylation sites on the RBD are shown in the amino terminal region. The brackets joining regions in RDB correspond to previously described disulfide bridges.

Spike protein has been described as a target of the humoral immune response, considering its localization on the viral particle and due to experimental evidence of its high antigenicity. The receptor binding domain (RBD) is located between R319 to F541 residues in the S protein, while the receptor binding motif (RBM) has been mapped between residues N437 to Y508, where no N or O-glycosylation sites have been described. Two non-glycosylated (NG) antigens were designed considering the non-glycosylated regions of RBD: NG19, located in S371-F541 and NG06, between S438-N487 (Figure 1).

The analysis for average antigenic propensity of NG06 was 0.9964 with two antigenic determinants (YNYLYRL and STEIYQAGS). For NG19, the average antigenic propensity was 1.0401 with seven antigenic determinants (ASASFSTFKCYGVSP, KLNDLCFTNVYADSFVIR, DFTGCVIA, YNYLYRL, STEIYQAGS, EGFNCYFPLQSYG, and VGYQPYRVVVLSFELLHAPATVCGP). Allergen prediction indicated that both sequences are probably not allergenic, as well as lacking epitope sequences against IgE, which justifies their safety as vaccine antigens. Additionally, we performed the analysis of the secondary structure (Figure S1) and determined the physicochemical properties of the NG06 and NG19 proteins (Table S2).

**Figure 2:**
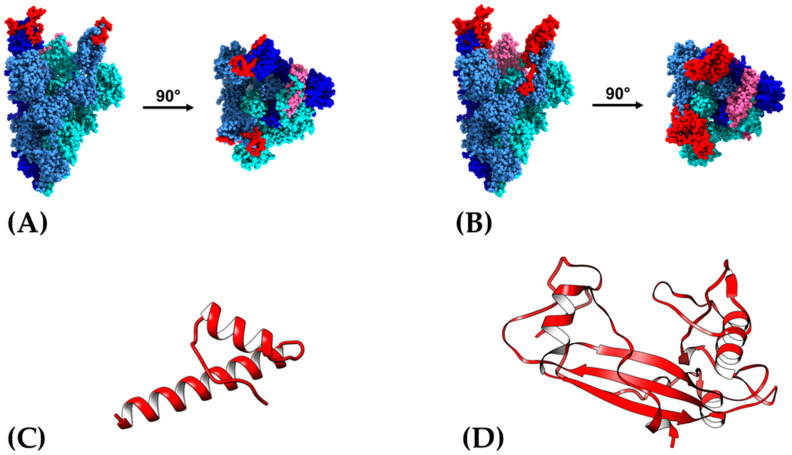
Structure of the NG06 and NG19 antigens. (**A**) NG06 region highlighted in the Spike protein, (**B**) NG19 region highlighted in the Spike protein. *De novo* predicted three-dimensional structure for NG06 (**C**) and NG19 (**D**) peptides. (**A**,**B**) Each monomer is shown in different shades of blue and the NG06 and NG19 regions within the RBD (open states RBD are showed in red and closed state in pink).

To theoretically visualize whether the selected NG proteins were located on the surface of the S protein, we calculated its tridimensional conformation employing the trimeric Spike protein at open state, which has been described as the accessible conformation to interact with the ACE2 receptor, based on the down and up positions of its RBD [43] (Figure 2). Panel A shows the location of NG6, a 90° turn locates the selected NG06 in the central region. Panel B shows NG19, also located on the apical region, but distributed on the surface and in the lateral Spike trimeric structure. Both NG06 and NG19 are hydrophilic, with a theoretical isoelectric point of 8.1 and 8.6, respectively, and a predicted high stability index (Table S2).

After structural modeling, the proteins were validated considering the Z value global quality graphs of the models obtained, as well as Ramachandran graphs. The Z values of both models were −3.99 for NG06 and −6.31 for NG19, which are within the calculated values for proteins resolved by NMR; also, local quality models do not exceed thresholds using window size values of 40 amino acids (Figure S2). Finally, in Ramachandran plots for NG06 protein, we observed 97.8% of the residues within the favored regions, 2.2% in additional permitted regions, while NG19 contains 90.6% amino acid in more favored regions, 8.1% in additional favored regions, and 1.3% in generously permitted regions. Both models presented 0% amino acids in non-favored regions, which indicates the good quality of the models obtained (Figure S3). The selected regions are shown within the Spike structure as regions highlighted in red, as part of each monomer in open (red) or pink (closed) conformation and as part of RBD (Figure 2A,B). Additionally, the final visualization of the *de novo* predicted models are shown in the panels (Figure 2C,D).

### 3.2. Antigen Expression in an Heterologous System

**Figure 3:**
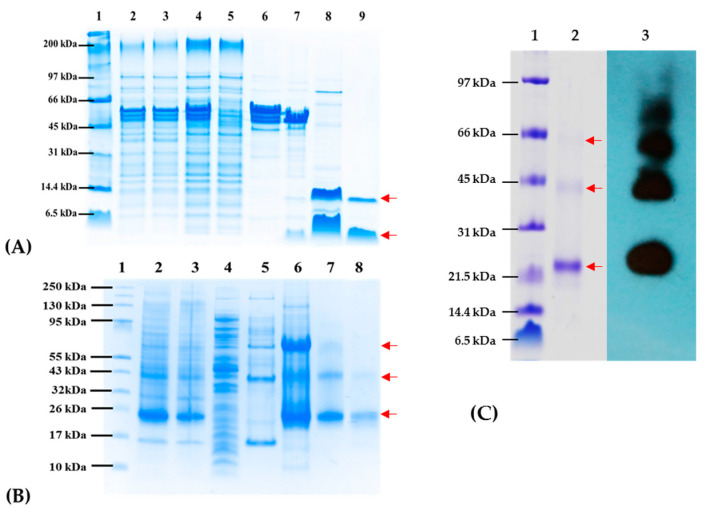
Purification steps of NG06 (**A**) and NG19 (**B**) and immunodetection by Western Blot (**C**). Line 1A, 1B, and 1C: Molecular weight marker, Line 2A and 2B: total fraction, Line 3A and 3B: insoluble fraction, Line 4A and 4B: soluble fraction, Line 5A and 5B: non-bound fraction obtained after purification by IMAC, Line 6A, and Line 6B: bounded fraction obtained after purification by IMAC, Line 7A: fraction bound after second IMAC, Line 7B: Refolding/SEC fraction, Line 8A: unbound fraction after second IMAC, Line 9A, 8B, and 2C: fraction obtained after SEC. Line 3C: Immunodetection by Western blot employing NG19 as antigen and NG06 immunized sera. Red arrows point to the purified, oligomeric antigens.

The expression vector pCri1a allows the expression of proteins translationally fused to the Maltose Binding protein (MBP), which increases the solubility of the resulting fusion peptide, and adding a histidine tag (His 6X) in the N-terminal region. MBP and His-Tag domain can be excised by digestion with TEV protease to obtain NG6 and NG19 peptides. Antigens expressed in *E. coli* were affinity-purified using nickel columns. Soluble, purified proteins were then digested with TEV protease and, finally, purified by Size Exclusion Chromatography (SEC). However, digesting with TEV protease did not proteolyze the MBP-TEV-NG19 fusion, so we decided to clone and express the NG19 antigen in the pCri8a vector that carries only a histidine tag. In this way, we were able to solubilize inclusion bodies, refold, purify the protein, and digest with TEV, this time using only His-TEV-NG19 to obtain the final NG19 antigen. Figure 3 shows Tricine-SDS-PAGE gels containing the chromatographic steps in purifying the antigens corresponding to the chromatograms obtained (Figures S4 and S5). NG06 and NG19 were able to form a homodimer under non-reducing conditions NG19 also showed a high propensity to form homo oligomers as we could verify it through *in silico* oligomerization approaches (Figure S6).

### 3.3. Immunization of Rabbits

**Figure 4:**
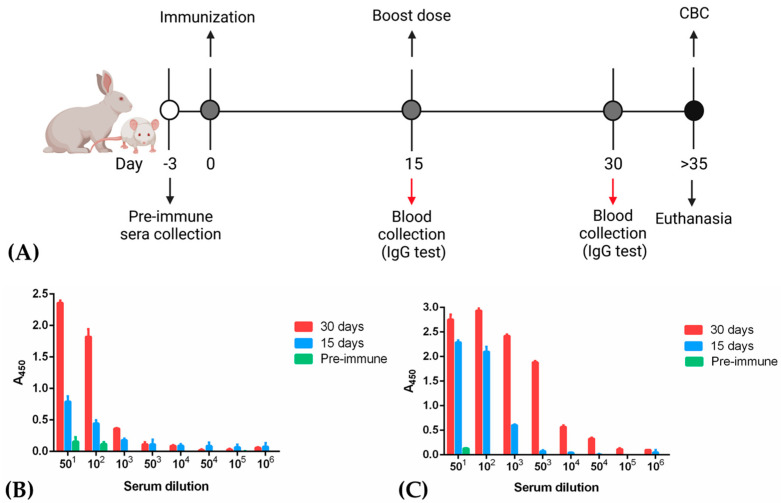
NG06 and NG19 proteins elicit immune responses. (**A**) Scheme of animal immunization with recombinant antigens. (**B**,**C**) Indirect ELISA titration of pre–immune and hyperimmune rabbit sera using antigens NG06 (**B**) and NG19 (**C**). CBC: Complete blood count.

Antigens were employed in immunization schemes for rabbit antibodies production (Figure 4A). NG06 and NG19 antigens were administered subcutaneously and intramuscularly in two female New Zealand rabbits. IgG quantification using immune plasma revealed recognition of recombinant antigens at a serum dilution of 1 to 10^6^; however, a higher titration was observed when NG19 was employed. Both antigens evoked a more efficient response when a second boost was employed (Figure 4B,C) with CBC values (Table S3). Furthermore, an ELISA assay was performed to assess antibodies specificity, using as antigens the NATtrol ™ Respiratory Panel 2 (RP2) Controls (Zeptometrix Corporation, Buffalo, NY, USA) respiratory pathogens panel and the 15 dpi and 30 dpi preimmune serum of the rabbits. The ELISA assay failed to recognize antigens present in the human respiratory extracts contained in the panel and were not statistically significant with respect to preimmune sera (data not shown). Additionally, we compared the affinity of theses polyclonal antibodies to several antigens, including native RBD protein; NG06 and NG19 antigens; showing an increased affinity in rabbit sera samples (Figure S7).

### 3.4. IgG from Recovered COVID-19 Patients Recognizes the Recombinant Antigens NG06 and NG19

**Figure 5:**
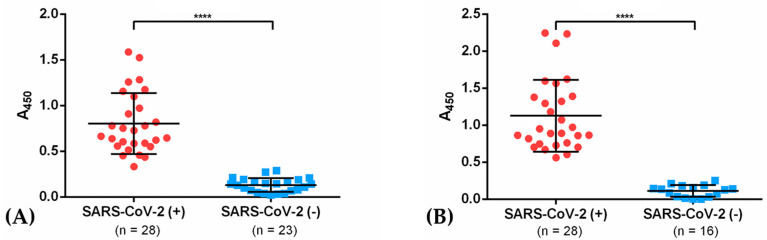
IgG response in SARS-CoV-2 positive patients and healthy subjects against the recombinant antigens NG06 (**A**) and NG19 (**B**). Data are presented as the mean of the triplicate OD values per subject. Each dot represents one subject and horizontal bars correspond to the mean values with SD. **** indicates a significance level of *p* < 0.0001.

IgGs from sera from recovered COVID-19 patients (2–4 weeks after recovery on average, 48% males, 52% females) showed affinity (at a 1:5000 dilution) to recombinant NG06 and NG19 antigens by indirect ELISA assays. For NG06, an A_450 nm_ = 0.80 ± 0.33 was observed in COVID-19 patients after recovery, and an A_450 nm_ = 1.13 ± 0.33 for NG19. In contrast, A_450 nm_ of 0.13 ± 0.08 and 0.11 ± 0.08 was obtained in the control group with both antigens. These results were consistent with those obtained with rabbit hyperimmune sera, where the sensitivity was also higher with the N19 antigen (Figure 5A,B).

### 3.5. IgGs Elicited after Immunization with Antigen NG19m in Mice Showed Neutralizing Activity in sVNT Assays

**Figure 6:**
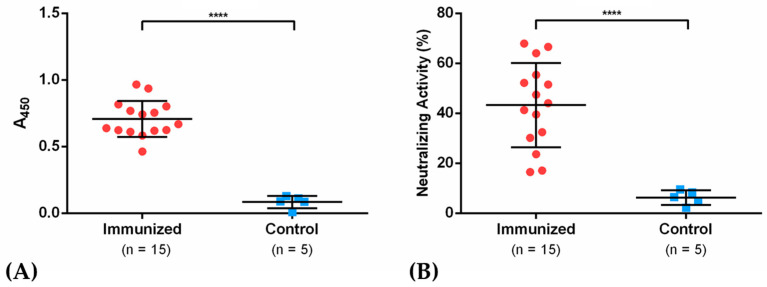
IgG antibody titers in mice sera (**A**) and neutralization activity through sVNT (**B**) in mice immunized with the NG19 antigen. **** indicates a significance level of *p* < 0.0001.

Considering the results obtained with sera from immunized rabbits and COVID-19 recovered patients, an immunization protocol was also performed in mice using the NG19 antigen. For this purpose, eight-week-old female Balb/c mice were immunized with 20 μg of antigen formulated with an aluminum hydroxide-based adjuvant approved for clinical use and after 15 days they were shot with a second booster dose at the same initial concentration (Figure 4A). The hyperimmune sera obtained 30 dpi was assayed in ELISA immunodetection and neutralization tests through sVNT assay. The data showed immunodetection of IgG from mice sera (Figure 6A) and neutralization values ranging from 17.1 to 66.9% in immunized mice against 2.1 to 9.6% in the control group (Figure 6B). These results indicate the potential neutralizing effect of the NG19 antigen in preventing the interaction of Spike protein and the receptor ACE2 in the presence of hyperimmune serum.

## 4. Discussion

The Spike protein of SARS-CoV-2, displays the open and close conformations, occurring in RBD, also referred as the down or up states, which are important for the binding of the S protein to the receptor ACE2. Thus, the inaccessible (closed) receptor state shows all the RBDs in the down position, covering the S2 subunits; while in the receptor-accessible (open) state, at least a single RBD in the trimeric S is in an up position, which is then turned outward from S2, thus exposing its receptor-binding surface [43]. Also, the region of the S protein with the most exposed epitopes comprises the apical zone of RBD domain in its open conformation [19]. We designed the NG06 and NG19 antigens in this region where the virus cannot exploit the glycan shield strategy or genetic drift without diminishing its fitness. In this way, the selected peptides may be effective as protective antigens if the virus continues to recognize the ACE2 receptor to mediate entry into cells. Also, most of the neutralizing antibodies previously described—such as CR3022 [44], DB-368-2 [45], 47D11 [46], B38, and H4 [47]—have been found mapping NG06 and NG19, with similar patterns for S protein from SARS-CoV, MERS-CoV, and SARS-CoV-2.

NG06 and NG19 lack of post-translational modification consisting in glycans, they could not evoke the production of autoantibodies produced in SARS-CoV-2 infection [48], targeting molecules such as host carbohydrates (eg, gangliosides) causing neuropathic Guillain-Barré or Miller Fisher syndromes [49,50,51]. In fact, a transverse myelitis case has been reported in a volunteer patient immunized against the SARS-CoV-2, where the protective agent was an adenovirus-based vaccine containing glycosylated S protein on its surface [52,53]. NG06 and NG19 could also be useful for immunodiagnostics since the presence of antibodies over these regions correlates with the presence of neutralizing anti-RDB antibodies that do not recognize glycan. These types of glycan-directed antibodies are common in autoimmune diseases or viral infections, such as cytomegalovirus, Epstein-Barr virus, hepatitis E virus, influenza A, and Zika virus [54,55].

A limitation of the antigens generally proposed as protective vaccines is that they do not encompass the viral variants of concern circulating in the human population. Also, several substitutions have been reported mapping the RBD present in the Spike protein. The vaccines candidates described in the present contribution NG06 and NG19 comprises the substitutions describe in the variants of concern described up to date: G476S, V483A, K417N, E484K, E484Q, N501Y, T478K, and L452R [56,57,58]. Indeed, considering the RBD-ACE2 is a protein–protein interaction, NG06 and NG19 antigens were designed to contain epitopes that can generate efficient immune responses, but also for their capacity to elicit neutralizing antibodies to block the interaction RBS-ACE2. the effect of these low-frequency substitutions on the immune response is so far unknown, and that these substitutions are limited geographically. In addition, vaccine antigens could be generated including the circulating variants in specific regions.

Among the available platforms for the production of recombinant antigens, *E. coli* possesses several advantages as its high growth rate, low nutritional requirements, availability of genetically-modified strains for easy scaling up the production process, and availability of vectors to facilitate the controlled protein expression [59,60]. Indeed, *E. coli* has been used as an expression system for vaccine antigens against different human viruses, including Zika virus, HPV, SARS-CoV, and SARS-CoV-2 [61,62,63,64]. Particularly for coronaviruses, there are examples in which the RBD of SARS-CoV produced in mammalian cells, insect cells and *E. coli* were compared, showing that all expression systems allow the production of protective antigens against SARS-CoV in mice [65].

The RBD domain expressed in *E. coli* has been shown to be capable of eliciting a cellular and humoral immune response leading to the production of neutralizing antibodies against SARS-CoV-2 [64]. However, lower neutralization titers are achieved using RBD compared to the S1 subunit of Spike protein [66] probably due to the use of unfolded and low-purity (60%) immunogens. Also, including glycosylation sites in an antigen based on a prokaryotic expression platform would lead to the exposure of absent epitopes in the native RBD domain, altering the immune response.

Soluble and high-purity NG06 was obtained through a fusion to MBP, subsequently digested with TEV protease and purified by IMAC and SEC. This strategy was unsuccessful for NG19 as we did not obtain enough antigen after TEV proteolysis. We hypothesize that in the three-dimensional structure of MBP-NG19 the cleavage site could be inaccessible due to steric hindrance [67]. Other approaches to ensure refolded and soluble expression include additional fusion partners, longer hinges between fusion partners and antigens, and the use of additives or purification strategies from inclusion bodies (IB). We also evaluated the expression of NG19 fused to GST and TrxA proteins (data not shown) unsuccessfully, in comparison to that observed for TrxA-RBD from SARS-CoV [63], due probably to differences in primary and secondary structure that could affect the solubility of RBD from SARS-CoV-2. We then decided to use IB to produce a refolded and soluble antigen employing previously described purification and refolding methodologies.

Rabbit immunization with RBD from SARS-CoV-2 has shown an improved production of high affinity IgGs with high neutralizing titers; 85% of the epitopes recognized by these RBD-elicited IgGs coincide with the antigenic domains selected for the antigens NG19 and NG06 described in this contribution [68]. Similar results have been obtained in another study where RBD was used as vaccine antigen in immunization assays involving non-human primates [69]. In addition, there are currently more than 10 RBD-based vaccine initiatives, some of which are already in clinical trials [70,71]. Our results demonstrate that both NG06 and NG19 can be recognized by specific IgGs from recovered COVID-19 patients. We also evaluated the neutralizing activity through sVNT of the sera of immunized mice and observed inhibitory activity in most of the samples. Finally, it should be emphasized that the use of antigens produced in *E. coli* reduces manufacturing costs, using already FDA-approved protocols, which are important factors in the current fight against SARS-CoV-2.

## Data Availability

Data is contained within the article.

## Supplementary Materials

The following are available online at https://www.mdpi.com/article/10.3390/vaccines9080928/s1; Figure S1: Prediction of secondary structure for NG06 and NG19 antigens. Figure S2: Validation of three-dimensional models of NG06 and NG19 using the Z-Score and local quality values. Figure S3: Ramachandran plots for validation of the *de novo* predicted structures for the NG06 and NG19 proteins. Figure S4: Chromatographic steps through FLPC for NG06 purification. Figure S5: Chromatographic steps through FLPC for NG19 purification. Figure S6: *In silico* oligomerization of NG19. Figure S7: Comparison of antigen affinity for NG recombinant proteins and native RBD. Table S1: Primer sequence employed in the present study. Table S2: Primary structure and physicochemical properties of vaccine antigen candidates. Table S3: Hematological values of the immunized rabbits on day 35.
