# Supplementary material for: Recombinant Antigens Based on Non-Glycosylated Regions from RBD SARS-CoV-2 as Potential Vaccine Candidates against COVID-19
Source: Vaccines (Basel). 2021 Aug 20;9(8):928. doi: 10.3390/vaccines9080928 (PMC8402574; doi:10.3390/vaccines9080928)
Supplement: Supplementary file 1 [file vaccines-09-00928-s001.zip › vaccines-1309729-supplementary.pdf]

Supplementary Material

Figure S1. Prediction of secondary structure for NG06 and NG19 antigens.

Figure S2. Validation of three-dimensional models of NG06 and NG19 using the Z-Score and local quality values.

Figure S3. Ramachandran plots for validation of the *de novo* predicted structures for the NG06 and NG19 proteins.

Figure S4. Chromatographic steps through FLPC for NG06 purification.

Figure S5. Chromatographic steps through FLPC for NG19 purification.

Figure S6. *In silico* oligomerization of NG19

Figure S7. Comparison of antigen affinity for NG recombinant proteins and native RBD

Table S1. Primer sequence employed in the present study.

Table S2. Primary structure and physicochemical properties of vaccine antigen candidates

Table S3: Hematological values of the immunized rabbits at day 35

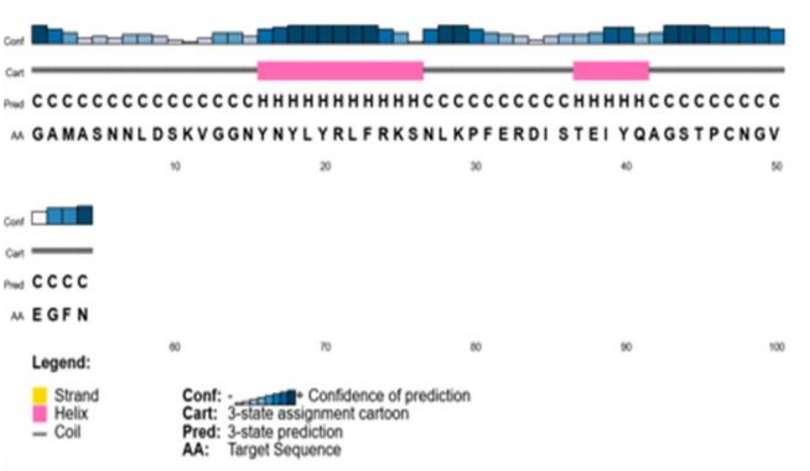

(A)

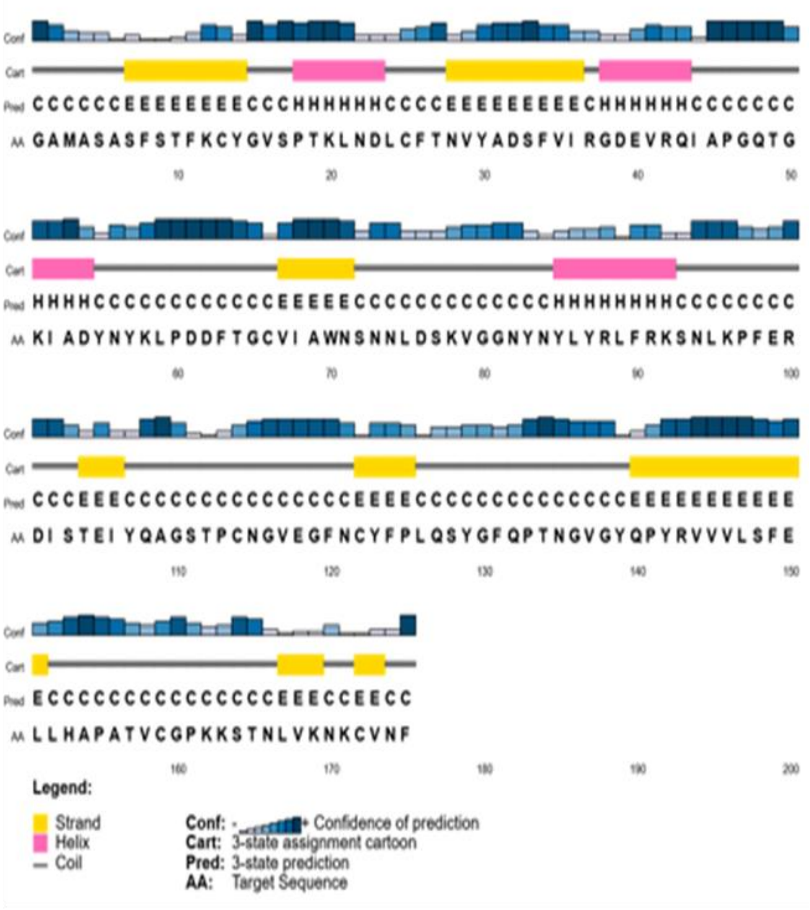

(B)

Figure S1. Prediction of secondary structure for NG06 (A) and NG19 (B)

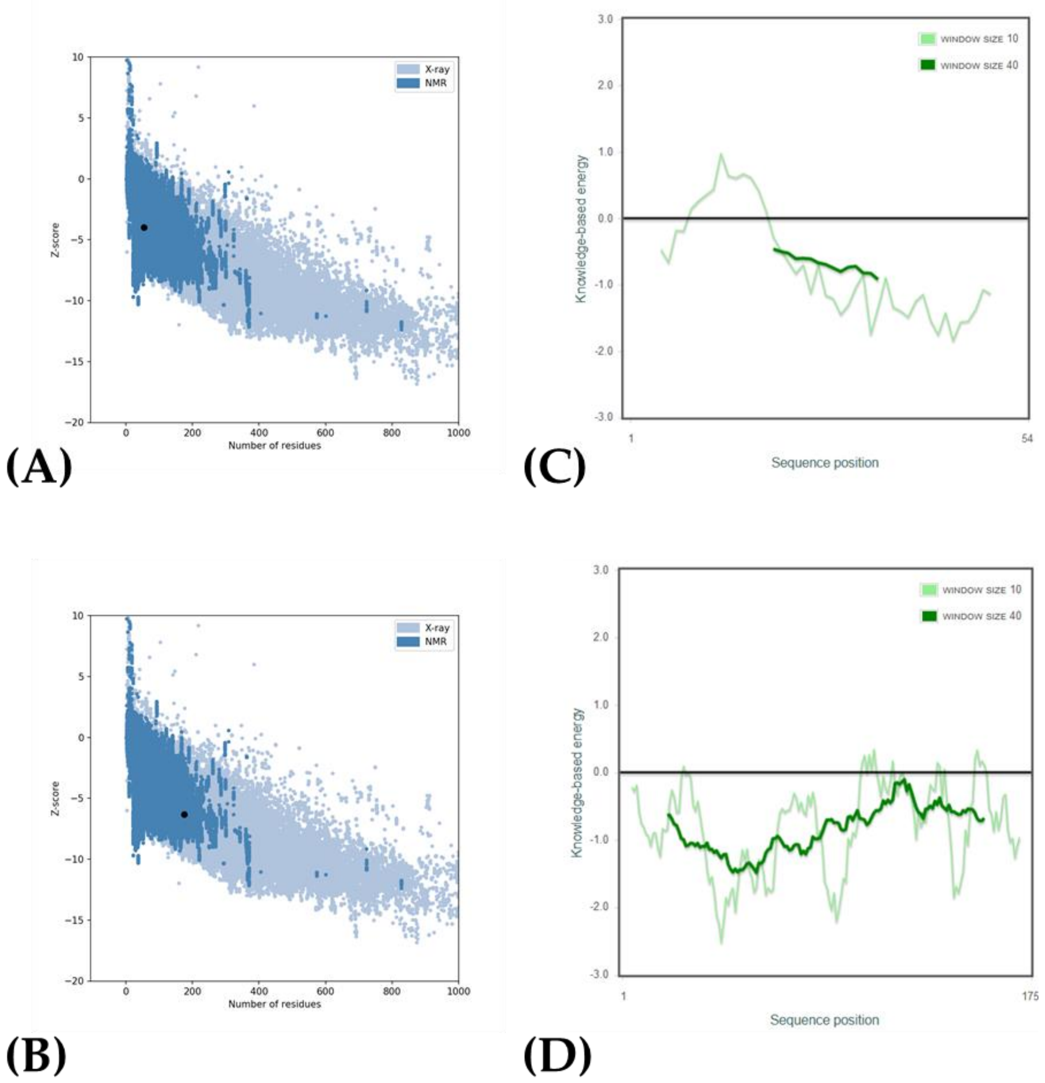

Figure S2. Validation of three-dimensional models of NG06 (A) and NG19 (B) using the Z-Score and local quality values for NG06 (C) and NG19 (D)

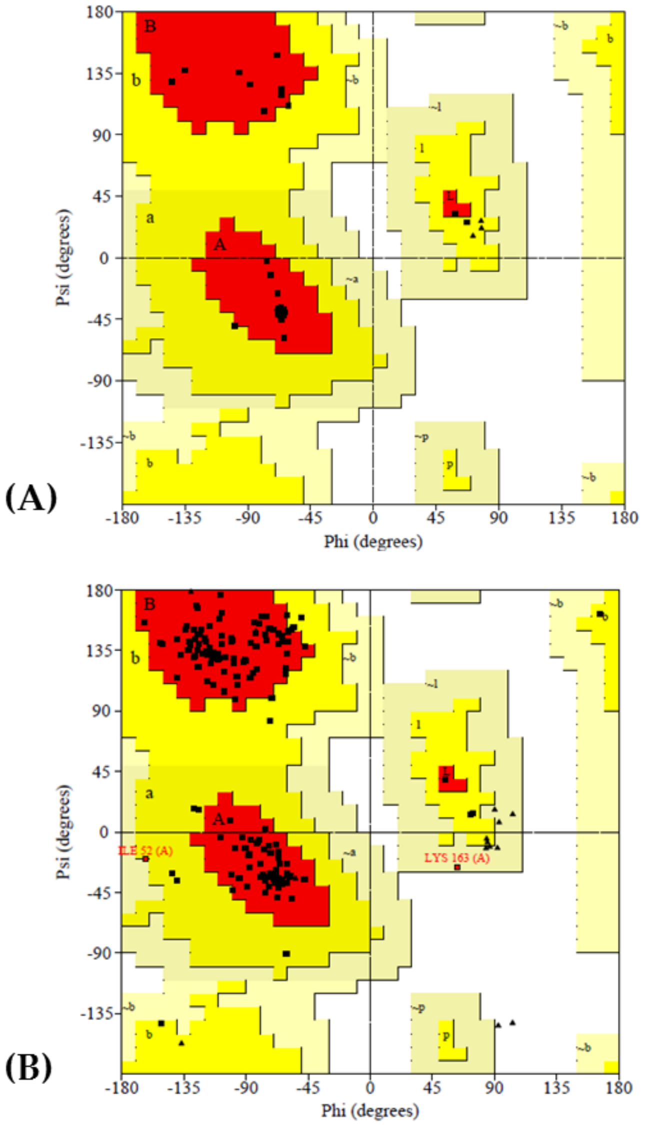

Figure S3. Ramachandran plots for validation of the de novo predicted structures for the NG06 (A) and NG19 (B) proteins

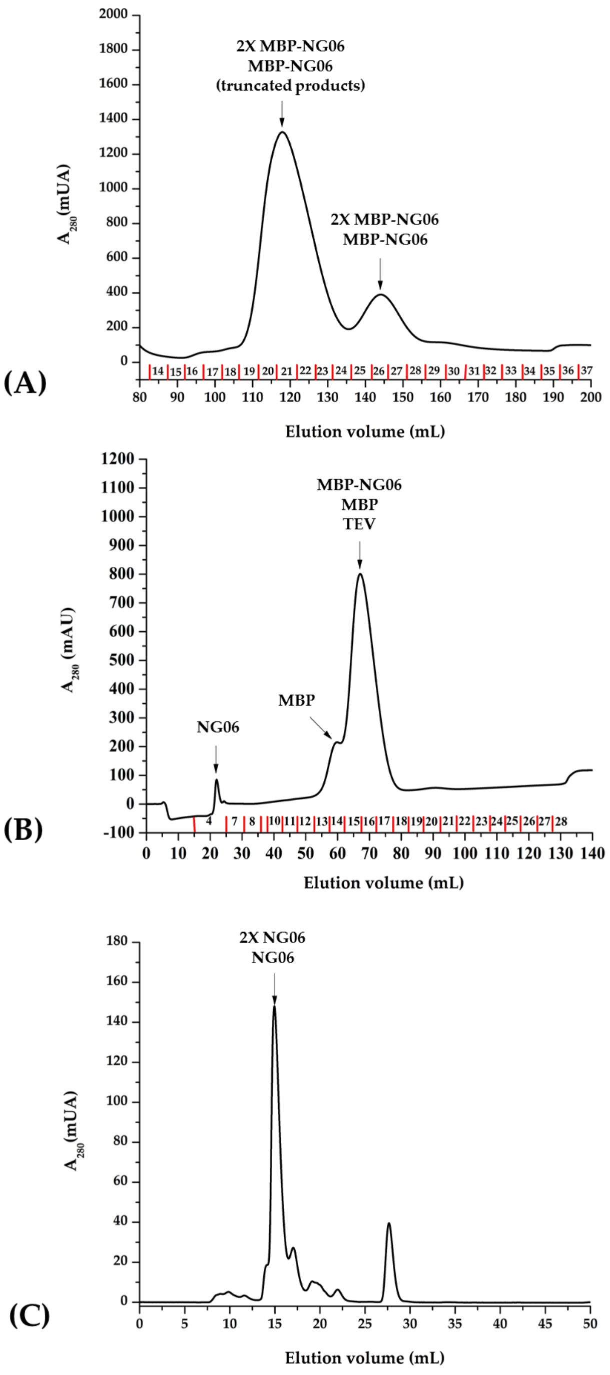

Figure S4. Chromatographic steps through FLPC for NG06 purification. A) Initial IMAC Chromatogram for NG06. B) Buffer exchange chromatogram for NG06 C) After TEV cleavage IMAC chromatogram for NG06. D) Final buffer exchange for NG06 purification

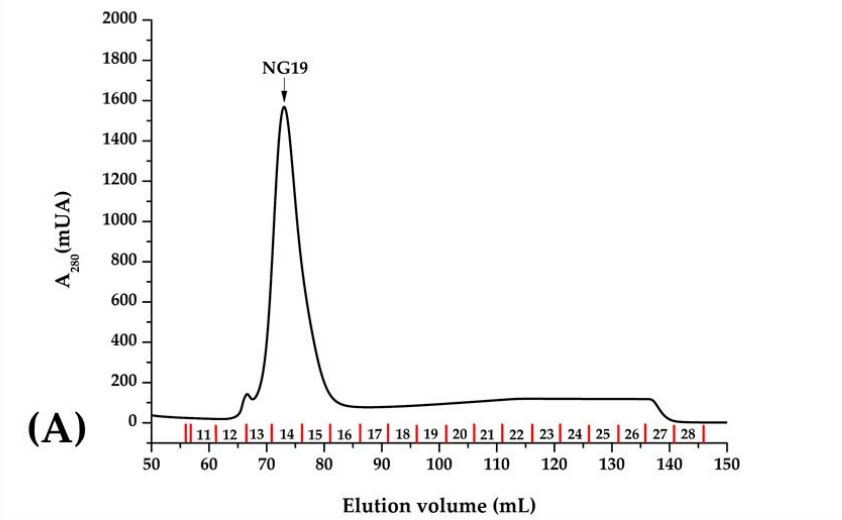

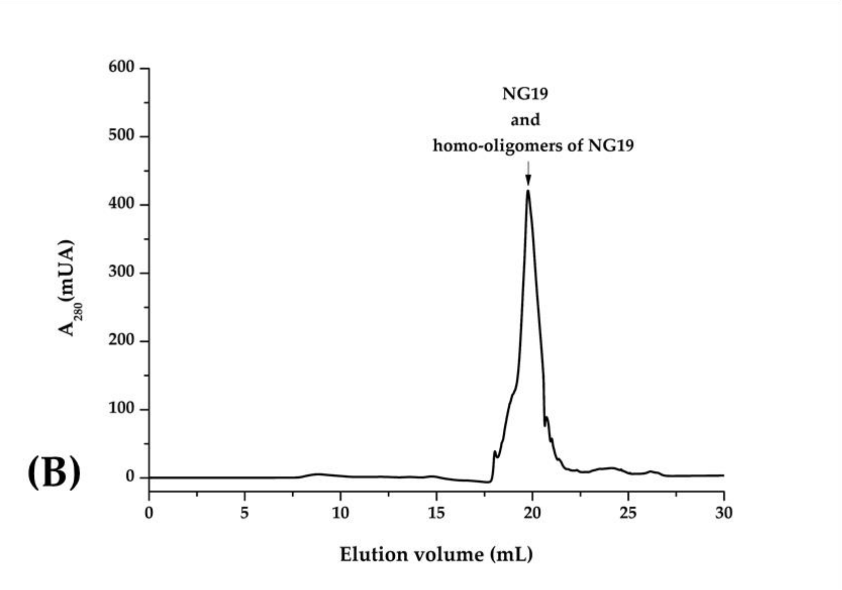

Figure S5. Chromatographic steps through FLPC for NG19 purification. A) Initial IMAC Chromatogram for NG19. B) SEC-Refolding chromatogram for NG19

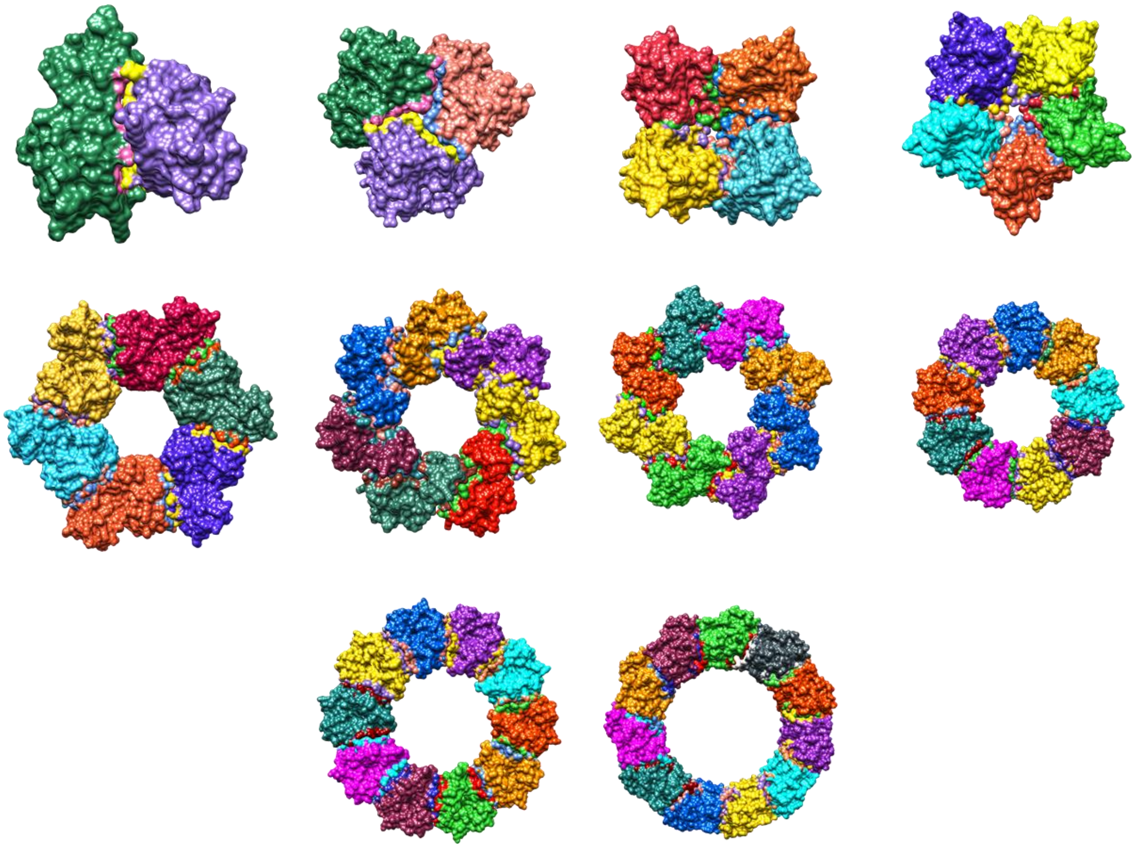

Figure S6: *In silico* oligomerization of NG19

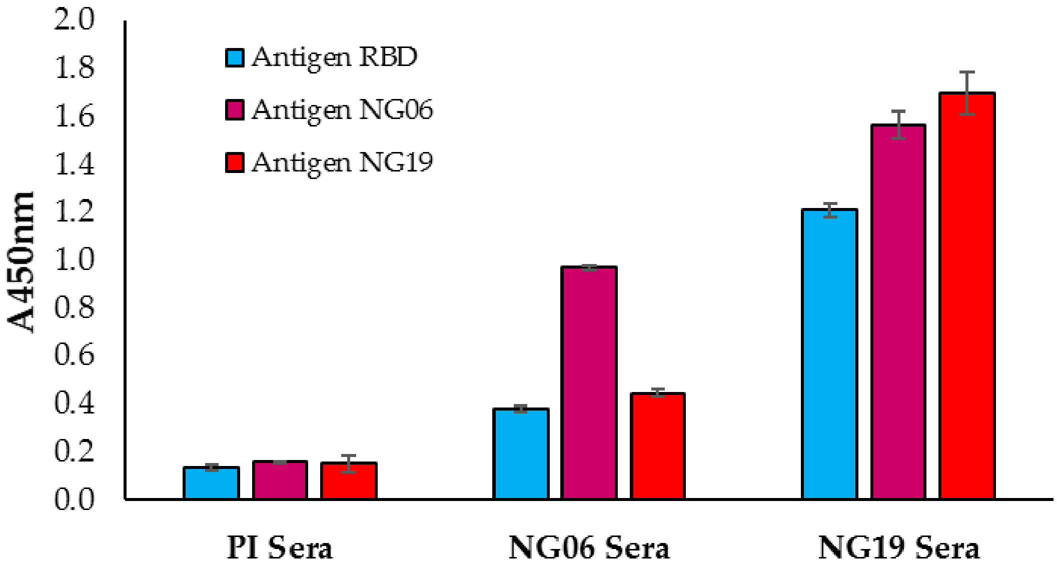

Figure S7: Comparison of antigen affinity for NG recombinant proteins and native RBD.

Table S1. Primer sequence employed in the present study

Primer Name	Sequence (´5→3´)
eNG06-F	AGCAACAACCTGGATAGCAAAGTCGGCGG
eNG06-STOP-R	TTAGTTAAAACCTTCAACGCCGTTACACG
eNG19-F	AGCGCGAGCTTCTCCACCTTCAAATGCTATG
eNG19-KWCEC-STOP-R	TTAGCATTCGCACCATTTGAAGTTGACGCATT
eNG06-STOP-R	TTAGAAGTTGACGCATTTGTTTTTGACCAGGT
NcoI-eNG06-F	CATG**CCATGG**CCAGCAACAACCTGGATAGCAAAG
HindIII-eNG06-R	CCC**AAGCTT**TTAGTTAAAACCTTCAACGCCGTTAC
NcoI-eNG19-F	CATG**CCATGG**CCAGCGCGAGCTTCTCCACCTTC
HindIII-eNG19-R	CCC**AAGCTT**TTAGAAGTTGACGCATTTGTTTTTG

* Restriction sites for NcoI and HindIII are shown in bold.

Table S2. Primary structure and physicochemical properties of vaccine antigen candidates

	NG06	NG19
Length (aa)	54	175
Molecular Weight (kDa)	6.0	19.4
Positive Charged Residues	6 (11.1%)	17 (9.7%)
Negative Charged Residues	5 (9.3%)	13 (7.4%)
Isoelectric Point	8.10	8.64
Instability Index	12.08 (stable)	16.64 (stable)

Table S3: Hematological values of the immunized rabbits at day 35

		NG06	NG19	Reference Values
**Hemogram**	**Hematocrit (L/L)**	0.45	0.41	0.34-0.50
	**Hemoglobin (g/L)**	144.00	123.00	98.00-158.00
	**Erythrocytes (×10^{12}/L)**	6.50	6.30	5.10-6.50
	**MCV (fL)**	69.00	65.00	57.80-80.30
	**MCHC (g/L)**	320.00	300.00	287.00-357.00
	**Platelets (×10^{9}/L)**	201.00	512.00	-
	**Proteins (g/L)**	60.00	70.00	60.00-75.00
	**Reticulocytes (%)**	1.70	2.10	0.00-3.00
**Diferential**	**Total Leukocytes (×10^{9}/L)**	6.60	19.80	5.00-12.00
	**Total Neutrophils (%)**	56.10	77.98	18.80-46.40
	**Band Neutrophils (×10^{9}/L)**	0.00	0.00	0.00-0.30
	**Metamyelocytes (×10^{9}/L)**	0.00	0.00	0.00-0.00
	**Myelocytes (×10^{9}/L)**	0.00	0.00	0.00-0.00
	**Lymphocytes (%)**	38.03	11.01	44.60-77.80
	**Monocytes (%)**	1.97	10.00	0.00-13.00
	**Eosinophils (%)**	0.00	0.00	0.00-2.40
	**Basophils (%)**	5.00	1.01	0.00-6.20
	**Nucleated eosinophils (/100 Leucocytes)**	0.00	0.00	0.00-0.00
	**Toxic neutrophils**	Negative	Negative	Negative
	**Atypical lymphocytes**	Negative	Negative	Negative
	**Erythrocyte morphology**	Normal	Normal	Normal
	**Anisocytosis**	Positive	Positive	Positive/Negative
	**Polychromasia**	Positive	Positive	Positive
	**Hemoparasites**	Negative	Negative	Negative
	**Dirofilaria**	Negative	Negative	Negative
	**Inclusion bodies**	Negative	Negative	Negative
	**Clumped platelets**	Positive	Negative	Negative
